# Dual-Inhibitors of N-Myc and AURKA as Potential Therapy for Neuroendocrine Prostate Cancer

**DOI:** 10.3390/ijms21218277

**Published:** 2020-11-05

**Authors:** Anh-Tien Ton, Kriti Singh, Hélène Morin, Fuqiang Ban, Eric Leblanc, Joseph Lee, Nada Lallous, Artem Cherkasov

**Affiliations:** Vancouver Prostate Centre, University of British Columbia, 2660 Oak Street, Vancouver, BC V6H 3Z6, Canada; aton@prostatecentre.com (A.-T.T.); ksingh@prostatecentre.com (K.S.); hmorin@prostatecentre.com (H.M.); fban@prostatecentre.com (F.B.); eleblanc@prostatecentre.com (E.L.); jlee@prostatecentre.com (J.L.); nlallous@prostatecentre.com (N.L.)

**Keywords:** prostate cancer, Myc, aurora a kinase, drug discovery, dual inhibitor, polypharmacology

## Abstract

Resistance to androgen-receptor (AR) directed therapies is, among other factors, associated with Myc transcription factors that are involved in development and progression of many cancers. Overexpression of N-Myc protein in prostate cancer (PCa) leads to its transformation to advanced neuroendocrine prostate cancer (NEPC) that currently has no approved treatments. N-Myc has a short half-life but acts as an NEPC stimulator when it is stabilized by forming a protective complex with Aurora A kinase (AURKA). Therefore, dual-inhibition of N-Myc and AURKA would be an attractive therapeutic avenue for NEPC. Following our computer-aided drug discovery approach, compounds exhibiting potent N-Myc specific inhibition and strong anti-proliferative activity against several N-Myc driven cell lines, were identified. Thereafter, we have developed dual inhibitors of N-Myc and AURKA through structure-based drug design approach by merging our novel N-Myc specific chemical scaffolds with fragments of known AURKA inhibitors. Favorable binding modes of the designed compounds to both N-Myc and AURKA target sites have been predicted by docking. A promising lead compound, 70812, demonstrated low-micromolar potency against both N-Myc and AURKA in vitro assays and effectively suppressed NEPC cell growth.

## 1. Introduction

Myc is a transcriptional regulator involved in numerous physiological and oncogenic functions due to its role as a central node in multiple signaling cascades regulatory networks [1]. Although Myc expression is highly regulated in normal cells, deregulated Myc can drive tumorigenesis by transcriptional programming of a large number of target genes, initiating and maintaining a large number of human cancers [2]. Numerous proto-oncogenes members in the Myc family (L-Myc, C-Myc, and N-Myc) are involved in the pathogenesis and progression of prostate cancer (PCa) [3,4].

Typical treatments for PCa involve targeting the androgen receptor (AR) directly either with abiraterone or enzalutamide [5,6]. However, patients undergoing AR-directed therapies for PCa can develop late stage castration-resistant prostate cancer (CRPC) tumors, eventually progressing to neuroendocrine prostate cancer (NEPC). As these patients gained resistance to therapies either by loss of AR-signaling dependence or AR expression [7,8,9], this highly aggressive and advanced stage of PCa has limited treatment options as even cytotoxic chemotherapy offers little survival benefit [10]. This change from the CRPC subtype to NEPC is observed by the amplification and overexpression of N-Myc [11]. In regular adult tissues, N-Myc is downregulated and not significantly expressed, but aberrant N-Myc amplification or overexpression occurs in approximately 40% of NEPC, leading to tumor aggressiveness, and suggesting N-Myc as a critical oncoprotein required for the development of NEPC [12]. The presence of N-Myc alterations has been associated with poor prognosis and outcome, consequently, development of metastasis is often observed shortly after the occurrence of N-Myc amplification [13]. Thus, N-Myc is an important driver of NEPC and could be considered as a desirable therapeutic target for potential treatment [14].

Overexpression and amplification of Aurora kinases has been observed in human cancers, such as PCa, and is often associated with poor prognosis due to their determinant role in cell transformation and oncogenesis [15,16]. In cancer, Aurora kinase A (AURKA) is overexpressed and involved in tumorigenesis by multiple mechanisms, such as phosphorylation of RAS-association domain family 1, to disrupt microtubules stabilization and cell cycle arrest, leading to unregulated proliferation [17]. In PCa, AURKA is highly expressed and further increased in CRPC, suggesting a critical role of AURKA in inducing therapy-resistance and disease progression [16,18]. Increased expression of AURKA has also been reported in precursors of most PCa [19]. These early findings support the therapeutic targeting of AURKA as a feasible approach in advanced CRPC and NEPC.

As AURKA interacts with different oncogenic pathways to favor cell proliferation, survival, and therapeutic resistance [20], it can also mediate Myc oncogenic effects in cancers. Simultaneous overexpression and activation of Myc and AURKA are commonly detected in NEPC and CRPC [21,22]. As N-Myc suppresses AR signaling and drives lineage plasticity, tumor aggressiveness, and AR-independent progression in PCa, AURKA interacts with N-Myc through protein-protein interactions (PPI) and forms a complex to protect N-Myc from Fbxw7-mediated proteasomal degradation [23]. As such, it was reported that simultaneous AURKA and MYCN gene amplifications were clinical indications of NEPC, especially in AR-negative NEPC, and both genes cooperate to induce neuroendocrine phenotype in prostate cells [22,24,25]. Therefore, potential therapeutics could exploit inhibition of both AURKA and MYCN alterations to treat PCa and NEPC.

There are currently no clinically approved compounds that can efficiently treat NEPC, hence there is a dire need for development of novel therapeutic approaches against NEPC. No compounds were designed to simultaneously interact directly with N-Myc and AURKA to treat NEPC. Since both were recently identified to be overexpressed in NEPC and the interplay between the two accentuate downstream oncogenic effects [12,21,25], we sought to design a novel inhibitor that could target both oncoproteins.

## 2. Results

To rationally design an N-Myc and AURKA dual-inhibitor, we first need to determine a potent scaffold against N-Myc specifically and another against AURKA. The second step to design a dual-inhibitor is to hybridize the active N-Myc scaffold and AURKA scaffold together to retain the potency against both N-Myc and AURKA.

### 2.1. Rational Design of Novel Potent N-Myc Inhibitors

#### 2.1.1. Homology Modelling of N-Myc-Max Heterodimer

We previously identified a potential pocket when C-Myc is associated to the homologous basic helix-loop-helix leucine zipper (bHLHLZ) domain of Max, forming a stable helical configuration which binds specifically to DNA E-boxes of targeted genes [26]. This target site is referred to as the DNA-binding domain (DBD). We screened this target site and initially identified compound 70063 as a potential lead to inhibit oncogenic effects engendered by Myc. We first sought to determine if this site was present in N-Myc.

**Figure 1:**
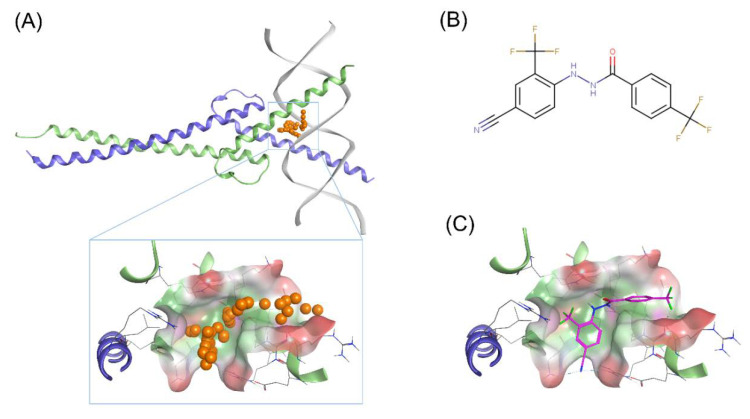
Proposed binding mode of 70551 into the N-Myc-Max DNA-binding domain (DBD) pocket. (**A**) Homology model of N-Myc-Max with the predicted binding site from MOE site finder (top) Max: green ribbons, N-Myc: blue ribbons, and DNA duplex: white. The dummy atoms in orange are used to represent the site, and potential occupancy for inhibition (bottom). Compounds occupying the same positions would clash with the DNA duplex. Hydrophobic surface: green, polar surface: pink, and exposed surface: red. The site is the same one as proposed in our previous publication with 70063. (**B**) 2D representation of the N-Myc specific lead, 70551. (**C**) 70551 in the proposed binding site of N-Myc-Max DBD. Ligand interaction network reveals one strong H-bond with Arg215 from 70551’s nitrogen group in its hydrazide linker.

As there is currently no crystal structure of N-Myc-Max heterodimer publicly available, a homology model for the DBD of N-Myc-Max was built based on the human C-Myc proto-oncogene protein sequence. Pairwise sequence alignment with the human C-Myc proto-oncogene protein revealed a similarity of 75.2% and an identity of 55.4% between the bHLHLZ regions of the two, corresponding to residues 377–464 for N-Myc and residues 350–439 for C-Myc. The similarity between the two is suitable to generate a homology model of the N-Myc bHLHLZ region. The model was built based on the publicly available 1.9 Å crystal structure of C-Myc-Max heterodimer bound to DNA (PDB ID: 1NKP). By using the induced fit approach, Molecular Operating Environment (MOE) generated an accurate homology structure to the crystal C-Myc, with a RMSD of 0.798 Å. As the human Max binding partner is similar in both N-Myc and C-Myc, the Max protein in 1NKP was added to the homology model. A binding site of identical composition and chemical character at the DNA interface of the N-Myc-Max model was identified, similar to the one we’ve identified previously in Figure 1A. In silico assessments show that the homology model’s score in PROSA was comparable to the crystal’s structure (MOE model: −2.44; 1NKP model: −2.55). Ramachandran plot analysis reveals that 96.5% of the homology model’s residues are located in favoured regions (Supplementary Table S1 and Supplementary Figure S1). Therefore, the homology model of N-Myc-Max DBD was used for docking simulations.

#### 2.1.2. Identification of Scaffolds Targeting the N-Myc-Max DBD Site

To determine a chemical starting point against N-Myc, we identified 70551 as potential Myc inhibitor from a series of Myc compounds developed in-house (unpublished results). 70551 is characterized by a hydrazide linker connecting the two substituted phenyl rings together as shown in Figure 1B. In the Myc-Max Luciferase transcription inhibition assay, 70551 reported a half maximal inhibitory concentration (IC_50_) of 4 μM. The docking pose of 70551 to the N-Myc-Max DBD reveals that the compound forms one strong H-bond with Asp215 of Max through its hydrazide linker, and a weaker H-bond with sidechain Lys419 of N-Myc through its cyano group. The cyano-trifluoremethyl phenyl group is involved in numerous hydrophobic contacts with N-Myc side chains of Leu397, Phe401, and Lys419, and with Max side chains of Arg212, Arg215 Ile218, Lys219, Phe222, and Arg239, anchoring VPC-70551 into its position in the DBD pocket as shown in Figure 1C. Therefore, we designated the N′-[4-cyano-2-(trifluoromethyl)phenyl]-4-(trifluoromethyl)benzohydrazide scaffold of 70551 as an active scaffold against N-Myc. Further characterization of 70551’s inhibitory profile in IMR32 (N-Myc overexpressing cell line) and HO15.19 (Myc negative cell line) to check for N-Myc specific inhibitory activity can be found further in the experimental results.

### 2.2. Structure-Based Drug Design of N-Myc and AURKA Dual-Inhibitors

**Figure 2:**
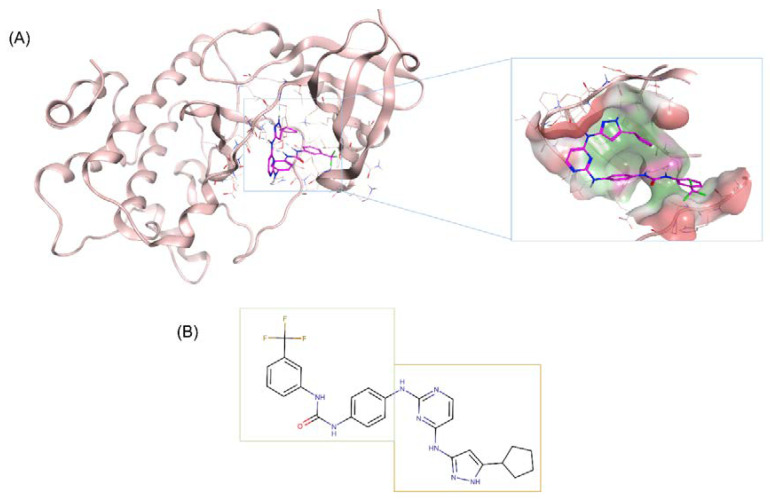
CD532’s binding mode to Aurora A kinase (AURKA). (**A**) CD532 (magenta sticks) binds to the active site of AURKA (pink ribbons). CD532 interacts with the ATP-binding hinge region, as well as with the protein-protein interactions (PPI) disruptor moiety, to induce conformation changes in AURKA (shown in the zoomed picture on right). Three-dimensional structure extracted from PDB 4J8M. Beta-sheets have been removed from the pocket for easier visualization of AURKA’s allosteric site. (**B**) CD532’s active scaffold can be divided into two main portions. One responsible for the N-Myc PPI disruption (encased in green), while the other acts as an ATP-competitor scaffold to bind to the hinge of AURKA (encased in yellow).

Based on the identified active N-Myc scaffold, we sought to merge it with the hinge binding scaffold of an AURKA specific inhibitor, CD532. CD532 is a novel conformation-disrupting AURKA specific inhibitor with high potency against AURKA (IC_50_ = 45 nM), and with strong degradation effects on N-Myc. The compound binds to AURKA and disrupts its PPI functions by stabilizing it in an inactive conformation [27]. The binding pose of CD532 in the crystal is shown in Figure 2A (PDB: 4J8M). CD532 can be divided into two sections: an aminopyrazole-pyrimidine acting as an adenosine triphosphate (ATP)-binding hinge region competitive inhibitor in AURKA, and a 3-trifluoromethyl-biphenyl urea acting as an AURKA/N-Myc interaction disruptor by inducing a global conformational shift in AURKA to weaken the AURKA and N-Myc protein complex and promote early proteasomal degradation of N-Myc [12]. As demonstrated by crystallographic data, the urea moiety of CD532 is responsible for stabilizing the biphenyl urea in a clashing orientation by contacting the catalytic Asp274, and the trifluoromethyl group forces displacements in the N-terminal domain of AURKA to disrupt binding to N-Myc. Thus, the tight binding mode of CD532 in AURKA shows that it can simultaneously inhibit AURKA kinase activity and disrupt stabilizing PPI with N-Myc. The hinge binding scaffold is presented in Figure 2B.

**Figure 3:**
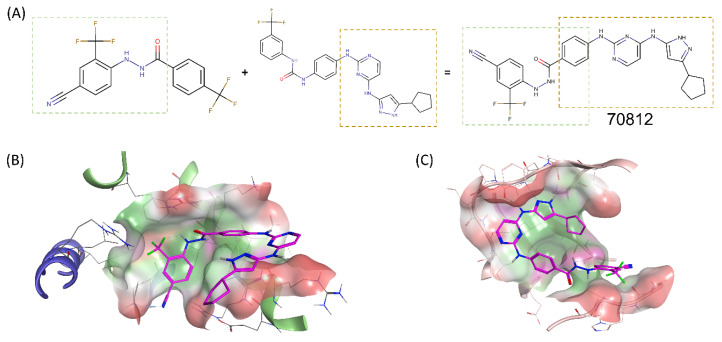
Merging of 70551’s scaffold with CD532’s scaffold for a novel dual-inhibitor. (**A**) The N-Myc specific scaffold, 70551 (left), shared a similar core with the AURKA specific scaffold, CD532 (right). Enclosed in green and yellow boxes are the sections of each scaffold responsible for the compounds’ inhibitory activity in their respective targets. The active parts of each scaffolds were merged by a commonly shared unsubstituted phenyl ring to generate 70812. (**B**) In N-Myc, 70812 (magenta sticks) has the critical H-bond with the backbone O of Arg215 of Max to maintain the scaffold in place in the DBD pocket. 70812’s tail is flipped towards the N-terminus of the DBD. (**C**) In AURKA, 70812 is docked in the ATP-binding core of AURKA and is tuck snuggly in the same position as CD532 in the AURKA pocket. Hydrophobic surface: green, polar surface: pink, and exposed surface: red. The beta-sheets have been removed for easier visualization of AURKA’s proposed binding site.

As the proposed N-Myc scaffold was similar to the PPI disruptor portion of CD532 (Tanimoto coefficient of 61.7%, as calculated with MACCS fingerprints), the two scaffolds were merged through a commonly shared phenyl ring to design novel inhibitor, 70812, as shown in Figure 3A. Accordingly, the proposed novel dual-inhibitors should maintain the N-Myc specific activity through the N′-[4-cyano-2-(trifluoromethyl)phenyl]benzohydrazide scaffold, and the AURKA specific activity through the aminopyrazole-pyrimidine ATP-mimetic scaffold. As CD532 targets only AURKA, leading to N-Myc degradation, we hypothesized that merging it with a N-Myc scaffold will yield a better direct dual-inhibitor of N-Myc and AURKA.

### 2.3. Binding Prediction of 70812 to AURKA and N-Myc through Docking

We sought to determine the possible binding modes of our dual-inhibitor and docked 70812 in our homology model of N-Myc and crystal structure of AURKA (PDB: 4J8M), as shown in Figure 3B,C. Analysis of 70812’s docking pose to AURKA demonstrate that the aminopyrazole-pyrimidine portion maintains the crucial hydrogen bond interactions with backbone residues Glu211 and Ala213 in the ATP-binding hinge region of AURKA. The cyclopentyl moiety interacts mainly through hydrophobic interactions with Leu139, Val147, Ala160, Leu161, Lys162, Leu194, Leu210, Leu263, and Ala273 and packs 70812 tightly into the hydrophobic pocket. The pyrimidine moiety is also maintained in place by a hydrophobic π−π interaction with sidechain of Gly216. In the PPI disruptor portion, although the hydrazide linker does not form H-bonds to Asp274 of the DFG motif, 70812 still interacts with it through its phenyl ring by polar contacts, and with Lys162 by its 4-cyano group. 70812’s N′-[4-cyano-2-(trifluoromethyl)phenyl]benzohydrazide is mainly interacting with AURKA’s PPI disruption site by an extensive hydrophobic interactions network with Leu139, Gly140, Lys141, Phe144, Gly145, Asn146, Val147, Leu162, Leu164, Thr217, Leu263, Val279, Ala281, and Pro282. Based on the predicted binding mode, 70812 interacts favorably with AURKA in a similar fashion to CD532 and could potentially induce a similar conformational shift to weaken and disrupt AURKA/N-Myc PPI.

70812’s binding mode to N-Myc is an interaction network that builds upon the one made by 70551. As it has the same scaffold, 70812 maintains the critical hydrogen bond to the backbone O of Arg215 on Max, and the 4-cyano group interacts with Lys419 of N-Myc through polar contacts. Notably, due to its extended tail, the compound adopts a pose that forces the phenyl ring to form hydrophobic π−π stacking with residues Arg212 and Arg215 of Max. The pyrimidine ring from CD532’s tail also forms hydrophobic π−π stacking with residue Arg212 of Max. Although the extended tail is flipped towards the N-terminus of the DBD and significantly exposed, a hydrophobic interaction network formed by Leu397, Phe401, and Lys419 of N-Myc, and Arg215, Ile218, Lys219, Phe222, and Arg239 of Max maintains 70812 in the N-Myc-Max DBD pocket. The tail also had hydrophobic contacts with Arg212 and Arg215 of Max to maintain 70812 in the pocket. Based on the predicted binding mode, 70812 interacts favorably with N-Myc in a similar fashion to 70551. Therefore, 70812 should exhibit inhibitory activity against both N-Myc and AURKA. The synthesis scheme for compound 70812 as provided by LifeChemicals, is presented in Appendix A.

To confirm the binding stability of 70812 with N-Myc-Max DBD and 70812 with AURKA, the protein-ligand complexes were submitted to extended 100-ns molecular dynamics (MD) simulations. The results of the MD simulations, as well as a summary of the protein-ligand contacts, are presented in Supplementary Figure S2. The stability of the interaction site was assessed based on the RMSD (root-mean square deviation) from the initial structure, calculated on C-α of the proteins and heavy atoms of 70812. Analysis of the trajectories shows that 70812 remains in the proposed N-Myc-Max DBD pocket. Although highly flexible initially, N-Myc-Max reached convergence at around 50ns. Comparatively, 70812 was stable from the beginning of the simulations mainly thanks to interactions with two residues: Arg215 and Arg239 of Max through a combination of H-bonds, hydrophobic and water bridges contacts. Throughout the simulation, hydrophobic interactions with Ile218 and Phe222 of Max, also participated in maintaining 70812 in the pocket. On the N-Myc side, residues Arg394, Leu397, Arg398 and Phe401 are the main residues involved in protein-ligand contacts with 70812. Similarly, analysis of the trajectories shows that 70812 remains in the AURKA ATP-binding site. The AURKA protein reached convergence at around 35ns while 70812 reached a stable RMSD at the beginning of the simulations. The fluctuations observed by 70812 is explained by the small movements of the compound in the large AURKA pocket. The protein-ligand contacts analysis shows that residues Glu211 and Ala213 of the ATP-binding core are involved in strong H-bonds with 70812 throughout all the simulation, as well as with Ala160, Tyr212, and Leu263, through hydrophobic contacts. The interactions between 70812 and the conformation disruption moiety are mainly hydrophobic and water bridges contacts, and include Leu139, Phe144, Val147, Lys162, Glu260, Asp274, and Ala281. Based on the results from the MD simulations, 70812 should bind to both N-Myc-Max and AURKA.

### 2.4. Experimental Validation of 70812 As Dual-Inhibitor

#### N′-[4-cyano-2-(trifluoromethyl)phenyl]-4-(trifluoromethyl)benzohydrazide as Active Scaffold against N-Myc

**Figure 4:**
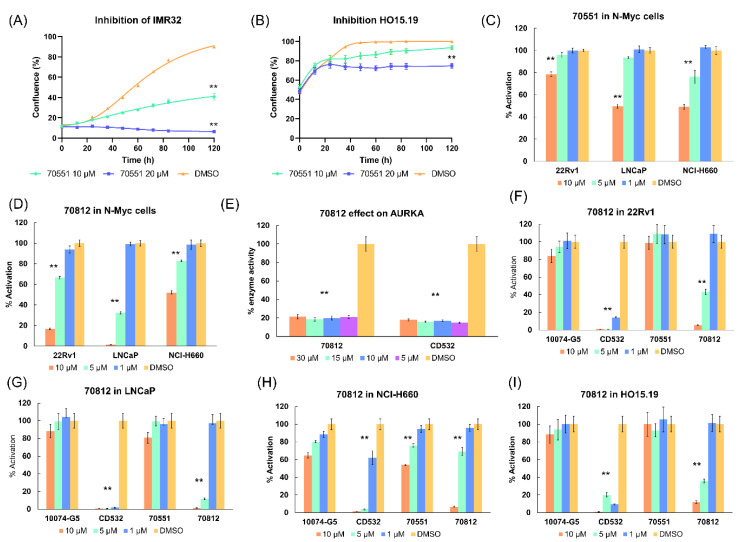
70812’s inhibitory profile against N-Myc and AURKA. (**A**) Inhibitory activity of 70551 was first determined. IMR32 cells were treated with 70551 at 10 µM and 20 µM for 120 h, and the growth was measured visually as a percentage of confluence (measure of cell proliferation on culture plate). (**B**) HO15.19 (Myc negative cell lines) were treated with 70551 at 10 µM and 20 µM for 120 h, and growth was determined visually as a percentage of confluence. 70551 was revealed to be active in positive control cell lines and showing acceptable toxicity in Myc negative cell lines. (**C**) Using an array of MTS assays, the specificity and potency of 70551 was further characterized in three N-Myc driven cell lines of LNCaP, 22RV1, and NCI-H660 at 10 µM, 5 µM, and 1 µM. (**D**) Inhibitory profile of 70812 in three N-Myc positive cell lines of 22RV1, LNCaP, and NCI-H660 at three concentrations of 10 µM, 5 µM, and 1 µM for 72 h as a measure of cell activity and growth. Cell density was measured using the PrestoBlue assay. (**E**) Reported AURKA enzyme activity following compound insertion at 30 µM, 15 µM, 10 µM, and 5 µM. 70812 had a similar inhibitory activity to CD532, at all concentrations tested. 70812 was assayed with its parental compound to determine its cell viability and profile in 22Rv1 (**F**) LNCaP (**G**), NCI-H660 (**H**), and HO.15.19 (**I**) at 10 µM, 5 µM, and 1 µM for 72 h. Cell density was measured using the PrestoBlue assay. All experiments were performed with N = 4 replicates. *p*-values were obtained using an unpaired *t* test against the vehicle control. Differences were considered significant when *p* < 0.005 (**).

It was first important to validate 70551’s activity and specificity. 70551 reported inhibitory activity in IMR32, a human neuroblastoma cell line in which N-Myc gene is amplified and actively expressed, at both 5 μM and 10 μM over 120 h, while it had minimal effect on the HO15.19, a N-Myc null cell line, although slight toxicity could be observed at 20 μM, as shown in Figure 4A,B. We then tested 70551 in N-Myc driven cell lines, 22Rv1, LNCaP, and NCI-H660, in an MTS assay at three concentrations of 10 μM, 5 μM, and 1 μM. Figure 4C shows that no significant inhibitory activity was detected at 1 μM in the three cell lines. At 10 μM, 70551 was able to inhibit cell viability principally in two cell lines (22Rv1 = 78.8% activation, LNCaP = 49.7% activation, and NCI-H660 = 49.2% activation), while inhibitory activity was minimal at 5 μM (% activation in cells >75% in 22Rv1, LNCaP, and NCI-H660). Therefore, we determined that the N′-[4-cyano-2-(trifluoromethyl)phenyl]-4-(trifluoromethyl)benzohydrazide scaffold of 70551 could be responsible for the observed potency of 70551 in N-Myc cell based assays.

### 2.5. Biological Characterization of 70812 as a Dual-Inhibitor

To determine the viability of 70812, we designed an array of assays to test its inhibitory properties on N-Myc driven cell lines and on AURKA kinase activity. Growth inhibition was determined in the same three N-Myc driven cell lines: 22Rv1, LNCaP, and NCI-H660. The inhibitor was then tested in HO15.19, a Myc negative cell line, to determine its toxicity profile. Therefore, compounds active in the three N-Myc driven cell lines and inactive in the Myc negative cell line are deemed to be able to target N-Myc specifically. Finally, to establish 70812’s AURKA selectivity profile, an adenosine diphosphate (ADP)-detection kinase assay was used to determine if the compounds could efficiently stop ADP being converted into ATP in AURKA. This set of assays allowed us to profile the proposed dual-inhibitor and its potential in directly targeting both N-Myc and AURKA.

#### 2.5.1. 70812 Is a Potent Inhibitor of Both N-Myc and AURKA

70812 had an IC50 of 2 µM in the luciferase reporter assay in LNCaP cells. Based on the promising inhibition activity of the compound, cell viability was further evaluated at concentrations of 10 µM, 5 µM, and 1 µM in the 3 N-Myc driven cell lines. No discernable inhibitory activity was detected in the three cell lines at 1 µM. At 10 µM, 70812 reported higher inhibitory activity in 22Rv1 (16.6% cell activation) and LNCaP (1.4% cell activation) than NCI-H660 (52.1% cell activation). Testing at 5 µM revealed similar inhibitory activity profiles, with the weakest activity observed in NCI-H660 (82.9% activation). Although 70812 had a stronger profile in LNCaP (32.5% activation), it remained weak in 22Rv1 (66.5% activation). Nonetheless, 70812 could inhibit N-Myc driven cell lines at low micromolar concentrations, as shown in Figure 4D.

To elucidate its AURKA inhibitory activity, we profiled 70812 by calculating the remaining % of AURKA enzyme activity when it was administered at four different concentrations of 30 µM, 15 µM, 10 µM, and 5 µM. Therefore, the more potent the compound, the less active AURKA should be. At all concentrations tested, 70812 had strong AURKA inhibitory activity (30 µM = 21.4% activity remaining, 15 µM = 18.7% activity remaining, 10 µM = 19.9% activity remaining, and 5 µM = 21.1% activity remaining), comparable to CD532 (Figure 4E). 70812 doesn’t show any concentration dependent activity in our assays as it exhibits similar highly potent activity against AURKA thanks to the ATP competitive moiety of CD532. Thus, both compounds behaved similarly at all micromolar concentrations tested. Based on the promising results from the AURKA-specific assay and N-Myc cell-based assays, 70812 was designated as a potential dual-inhibitor of both N-Myc and AURKA.

#### 2.5.2. 70812 Reduces Growth of LNCaP and 22Rv1 Cells in a Dose-Dependent Manner

The anti-N-Myc potency of 70812 and its effect on cell proliferation was compared against its parental compound (70551), CD532, and the Myc control, 10074-G5. Compounds were evaluated in an MTS assay using 22Rv1, LNCaP, and NCI-H660, and cell viability was assessed after 72 h of incubation with the tested molecules at three initial concentrations of 10 µM, 5 µM, and 1 µM. Figure 4F–I show that 70812 is a more potent inhibitor, compared to 70551 and 10074-G5, in 22Rv1, LNCaP, and NCI-H660 cells, at all concentrations tested, thanks to its dual-inhibition properties. While it seems that CD532 is more potent than 70812, its activity could be related to its cytotoxicity, as observed in the N-Myc negative cell line, HO15.19.

**Figure 5:**
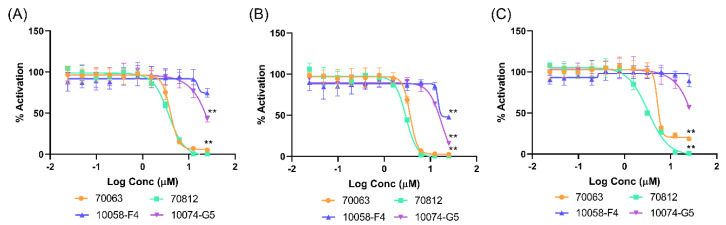
70812’s IC_50_ in N-Myc driven cell lines. The N-Myc inhibitory activity of compound 70812 in comparison to 70063, 10058-F4, and 10074-G5 in 22Rv1 (**A**)**,** LNCaP (**B**), and HO15.19 (**C**), administered through serial concentration. All experiments were performed with N = 4 replicates. *p*-values were obtained using an unpaired *t* test against the vehicle control. Differences were considered significant *p* < 0.005 (**).

Moreover, 70812 administered in serial dilution (Figure 5A–C) indicates that 70812 potently inhibits the growth of 22Rv1 and LNCaP cells with IC_50_ of 3.71 µM and 3.05 µM, respectively, while 10058-F4 and 10074-G5 were ineffective even at 10 µM, demonstrating its strong N-Myc specific activity. However, due to the central role of N-Myc and AURKA in cells, general toxicity should be expected for the compound; therefore, the reported toxicity is proportionate with its inhibitory activity in N-Myc driven cell lines.

**Figure 6:**
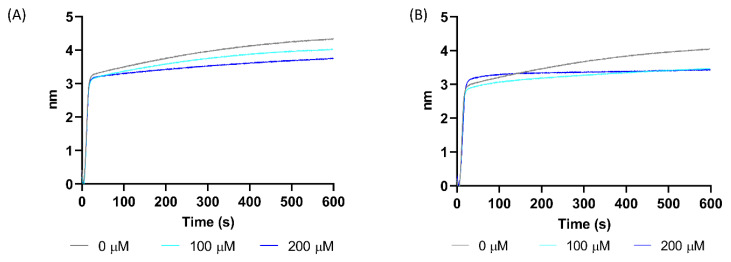
70812 disrupts DNA binding to N-Myc-Max DBD. (**A**) Inhibition of Myc-Max interaction by 70551 with biotinylated E-box was quantified by bilayer interferometry (BLI) at concentrations of 0 µM, 100 µM, and 200 µM. (**B**) 70812 could disrupt DNA from binding to the N-Myc-Max DBD at concentrations of 100 µM and 200 µM in BLI assays. The experiments are representative of 3 biological replicates.

This increase in potency compared to 70551 (22Rv1 IC_50_ = 15.6 μM and LNCaP IC_50_ = 11.1 μM) can be partly attributed to better disruption of the N-Myc-Max DBD binding to DNA E-boxes. Although 70551 is active in N-Myc specific cell-based assay, bio-layer interferometry (BLI) binding assays which measure direct interactions between DNA and the N-Myc-Max DBD complex, indicated that 70551 demonstrated weak disruption of DNA binding to the Myc-Max DBD pocket, as shown in Figure 6A. Comparatively, BLI experiment on the N-Myc-Max DBD was performed to characterize the mode of action of 70812, and, at the two concentrations tested, it can effectively disrupt DNA binding activity to the N-Myc-Max heterocomplex, thus inhibiting N-Myc-dependent downstream activity. (Figure 6B).

## 3. Discussion

Considering that Myc is central to the development of a broad range of adult organs, blocking Myc transcription factors might systemically trigger devastating and irreversible side effects [28]. However, Myc inactivation could be a viable therapeutic strategy as intermittent Myc inhibition was effective at containing growth of KRas-driven lung tumors in experimental mouse models [29].

Targeting Myc through conventional structure-based drug design approaches is challenging due to the intrinsically disordered proteins nature of Myc and its obligate partner, Max, and the lack of distinct pockets on the surface of either protein [30]. Nonetheless, there are a few prominent compounds, such as 10058-F4, 10074-G5, JY-3-094, KJ-Pyr-9, and Mycro3, directly inhibiting Myc dimerization with Max, demonstrating the potential of therapeutic targeting Myc [31,32,33,34,35,36]. One of the first preclinical compound identified through high-throughput screening, 10058-F4, and its analogue 10074-G5, were shown to bind directly to Myc’s bHLHLZ (basic helix-loop-helix leucine zipper) domain to disrupt formation of the Myc-Max heterodimer, but only had modest anti-proliferative effects. An optimized derivative, JY-3-094, had stronger ability to disrupt the dimerization between Myc and Max proteins, but was limited to high µM IC_50_ [37]. These small molecules have shown to be effective in cells by inhibiting the interaction between Myc and Max and interfering with Myc transcriptional activity. Other compounds, such as Mycro3 and KJ-Pyr-9, target the interaction between Myc-Max and blocks the formation of the Myc-Max-DNA complex and were active in Myc-amplified human pancreatic and breast cancer cell xenografts [33]. Compounds, such as KSI-3716, demonstrated that successfully blocking complex formation of N-Myc-Max with DNA [38,39] would cease transcription of target gene promoters that drives tumorigenesis in bladder cancer, suggesting inhibition of N-Myc as a possible therapeutic approach [40].

As Aurora kinases represent potent cancer targets due to their functional diversity, numerous AKIs (Aurora kinase inhibitor) have been proposed to suppress cell proliferation, migration, invasion in cancer cells, and to inhibit the progress and growth of many cancers. MLN8237 (Alisertib) and its predecessor, MLN8054, are AURKA specific small molecule inhibitors and displayed antiproliferative activity in numerous human tumor cell lines, including lung and prostate. MLN8054 and MLN8237 disrupt stabilization of N-Myc by AURKA, by inducing conformational shifts away from the kinase active state, leading to N-Myc degradation, and suppression of tumor growth [41]. MLN8237 recently underwent stage II clinical trials for NEPC treatment [21]. However, as they were designed as ATP-competitive inhibitors of AURKA, they exhibited modest effects on N-Myc-amplified neuroblastoma cells. Consequently, patients with N-Myc-amplified neuroblastoma responded weakly to MLN8237 treatment, underlying the possibility that MLN8237 may not be inhibiting N-Myc sufficiently [42]. Other Aurora kinase specific small molecules such as VX680 and VX689, target the ATP-binding pocket of Aurora kinases and have demonstrated potent anti-tumour activity in PCa cell lines [16,43]. Although VX689 was undergoing clinical trial phase II for leukemia, patients showed toxic adverse effects following administration, and was subsequently terminated. Similarly, CYC-116 and PHA-739358 (Danusertib) are pan-Aurora kinase inhibitors with potent anti-proliferative activity against cancer tumors, including PCa. While CYC-116 was terminated in phase 1 trial, Danusertib, although well tolerated by patients with advanced or metastatic solid tumors, only showed minimal efficacy in patients with metastatic CRPC [44,45]. Unfortunately, none of the proposed AKIs have been approved for clinical use yet due to many of them showing adverse effects in clinical trials, highlighting the difficulty in designing inhibitors with high specificity and potency to Aurora kinases [46].

In our previous study, we addressed the development of a new type of anti-Myc compound series for PCa treatment by targeting the Myc-Max heterodimer DBD pocket [38]. In this present study, we utilize computer-aided drug design methods to develop a novel dual-inhibitor, 70812, targeting both the N-Myc-Max DBD and the AURKA ATP binding region. The compound is an enhanced version of a Myc-specific compound, 70551, to which we’ve attached the AURKA-specific scaffold from CD532. With this additional functionality, the compound could also potentially disrupt the stabilizing interaction between AURKA and N-Myc. These triple inhibitory functions of 70812 lead to a better-defined inhibitory profile over our previous Myc compound and be used as chemical starting point for dual-inhibitor design against NEPC.

We first constructed a homology model of the N-Myc-Max DBD complex, using C-Myc as template (PFB ID: 1NKP [47]). We identified 70551 as a potent N-Myc inhibitor from our series of Myc compounds (unpublished results) and docked it to our homology model. Our binding model revealed features for a hit compound with an active scaffold consisting of a N′-[4-cyano-2-(trifluoromethyl)phenyl]-4-(trifluoromethyl)benzohydrazide. Thus, we identified an active scaffold against N-Myc driven cell lines.

CD532 is a novel AURKA inhibitor that was designed mainly as an ATP competitive inhibitor in AURKA’s hinge region. Although CD532 was considered a weaker inhibitor of AURKA compared to MLN8237, its potential as a conformation disruptor to destabilize AURKA in an inactive form, revealed further possibilities for drug design of targeting both the kinase activity and PPIs of AURKA. Based on the structural information of CD532 and its binding pose to the DFG-in form of AURKA, we observed that the PPI portion of the compound, the biphenyl urea moiety, shared similar features to the scaffold we designed for N-Myc-Max DBD. By using the commonly shared phenyl ring between the AURKA and N-Myc scaffolds, we computationally designed and subsequently synthesized compound 70812 as potential dual-inhibitors against NEPC.

In AURKA, 70812 is docked in the ATP-binding core of AURKA and interacts with Glu211 and Ala213 of the ATP core. 70812 is tuck snuggly in the same position as CD532 in the AURKA pocket, interacting with Asp274 of the DFG motif. In N-Myc, 70812 maintains the critical H-bond with the backbone O of Arg215 of Max to keep the scaffold in place in the DBD pocket, and its AURKA tail is flipped towards the N-terminus of the DBD. Extended MD simulations support the proposed interaction networks that maintain 70812 in the pocket of both proteins. MD simulations also show that the ligand does not disrupt the structural integrity of the N-Myc-Max heterodimer and indeed acts by binding to the DBD pocket.

Based on the docking pose of 70812 in N-Myc and AURKA, it should exhibit inhibitory activity in both N-Myc and AURKA. In the LNCaP luciferase transcription assay, 70812 reported IC_50_ values of 2 µM, compared to the IC_50_ of 4 µM of the parental 70551. 70812 was potent in inhibiting AURKA’s activity, which can be extrapolated by its binding mode. Steric clashes of 70812’s moieties shift the activation loop in an inactive orientation, as well as moving the N-terminal domain away from its stable and active position for PPI with N-Myc. However, the possibility that 70812 exerts allosteric effects by binding to other sites on either AURKA or N-Myc should not be dismissed. Although there’s a lack of distinct pockets on the surface of N-Myc, recent novel covalent inhibitors have been shown to bind directly to the disordered regions of Myc [48]. Similarly, the presence of distinct pockets along the surface of AURKA, raises the possibility of allosteric effects for 70812. Nonetheless, based on the current predicted binding poses, we concluded that to maintain dual-inhibitor properties, 70812 must bind in the AURKA PPI disruptor pocket, and the N-Myc-Max DBD site.

The activity of 70812 was further illustrated by its ability to inhibit growth in PCa and NEPC cell lines. The selected cell lines LNCaP, 22Rv1 and NCI-H660, are characterized by MYCN gene amplification and overexpression. While LNCaP and 22RV1 are considered classical human PCa cell lines, as they are directly derived from human PCa patients, only the NCI-H660 cell line is considered as a genuine NEPC cell line model. As NCI-H660 is AR-negative and overexpresses AURKA and N-Myc, this cell line represents an important preclinical model from which to determine the potential of our dual-inhibitor against NEPC.

With the additional AURKA inhibitory tail added to the N-Myc scaffold, we only observed slightly improved potency of 70812 against PCa cell lines tested compared to 70063 from our previous study. The lead dual-inhibitor, 70812, demonstrated anti-proliferative activity in the low micromolar range in N-Myc driven cell lines (IC_50_ of 3.71 µM in 22Rv1 and 3.05 µM in LNCaP). 70812 had an improved inhibitory profile in NEPC cell lines compared to all tested compounds, especially its parental compound of 70551. Thus, it is possible to consider that 70812’s NEPC inhibitory activity is derived from its anti-AURKA and anti-N-Myc profile. To note, based on our compounds, substitutions on the proposed N-Myc scaffold could increase specificity against N-Myc. Analogues of 70551 should be further explored to determine the full range of substitutions to increase potency.

Therefore, the inhibitory profile of 70812 can be traced back to mechanisms proposed in this study, which is not necessarily the case for active compound 70551. 70812’s activity is significantly enhanced compared to 70551 by adding the AURKA warhead tail. Since 70812 is targeting two key proteins essential in cell proliferation and survival, it is expected that the compound would exhibit certain cytotoxic properties. Nonetheless, by interfering simultaneously with multiple disease pathways, 70812 has a higher probability of hitting multiple nodes in the NEPC network for more efficient inhibitory effects. Thus, there is possibility of exploring 70812 as a multi-purpose cytotoxic chemotherapy agent, yet the compounds would require further toxicity class evaluation [49,50].

## 4. Materials and Methods

### 4.1. In Silico Experiments

#### 4.1.1. Homology Modelling of N-Myc-Max Heterodimer

The human N-Myc proto-oncogene protein sequence (UniProtKB ID: P04198) and C-Myc proto-oncogene protein sequence (UniProtKB ID: P01106) were retrieved from UniProtKB database [51]. MOE-Align, available from the Molecular Operating environment suite of programs [52], was used to generate pairwise sequence alignment between the two sequences. Homology model was built based on the publicly available 1.9 Å crystal structure of C-Myc-Max heterodimer bound to its DNA partner (PDB ID: 1NKP [47]). The homology modeling algorithm within MOE was used to generate the model of N-Myc. Human Max binding partner (UniProt ID: P61244) is similar in both N-Myc and C-Myc; therefore, the Max protein in 1NKP was merged in the N-Myc-Max heterodimer homology model. Based on the high identity between C-Myc’s and N-Myc’s bHLHLZ, the N- Myc target site was similar to the one where 70063 binds into the pocket of the C-Myc-Max DBD [38]. MOE homology modelling offers the possibility of generating a model through induced fit by including 70063 as a bound ligand in the structural template to increase the accuracy of our model [53]. Computational evaluation of the homology model’s protein geometry was carried out with MOE’s phi-psi plot tool, as well as with ProSA-web [54].

#### 4.1.2. Protein and Ligand Preparation

The homology model of the N-Myc-Max DNA binding pocket was prepared using the Protein Preparation Wizard within Maestro 9.3 suite from Schrödinger LLC (New York, NY, USA) [55]. The protocol ensures adequate addition of hydrogen atoms and reassignment of bond orders, before submitting the protein structures to energy minimization with OPLS3e forcefield until the RMSD relative to the starting geometry reached 0.3 A [56]. Using MOE, ligands were prepared through the “wash” process to protonate or deprotonate them. The ligands were then minimized with the Merck molecular force field 94x (MMFF94x) force field.

#### 4.1.3. Molecular Docking

The Glide docking program from Schrödinger LLC was used to perform docking analysis of compounds 70551 and 70812 [57]. The docking grid was defined as a 20 Å box centered on the binding region of 70063 to the Myc-Max DBD structure. The compounds were docked using Glide SP (Standard Precision) mode with all other settings set to default. The docking poses were then ranked by glidescore, and the highest scoring pose was selected as the most probable binding mode between compounds and protein targets. Docking poses with low binding affinities (high glidescore values) were discarded as potential binding modes. Docking to AURKA was performed with PDB structure 4J8M with the docking grid defined around CD532.

#### 4.1.4. Molecular Dynamics Simulations

Molecular dynamics simulations were carried out with the Desmond package from Maestro [58]. 70812 complexed with our N-Myc model was centered in an orthorhombic box with equidistant buffer. The simple point-charge (SPC) solvent model was used to add water molecules to the system. Chloride and sodium ions were added to neutralize the net charge of the solvated system. Salt were added to a concentration of 0.15 M to maintain protein structural stability. The system was then minimized for 100 ps. The system ran for 100 ns in a NPT ensemble set at 300 K with the applied OPLS3e force field [59]. Atomic coordinates were recorded every 10 ps during the simulations. Output trajectories were then analyzed with Maestro. The same simulation was carried out with the 70812-AURKA complex.

### 4.2. In Vitro Evaluation of Hit Compounds

#### 4.2.1. eGFP Cellular Myc Transcription Assay

The eGFP Myc transcriptional activity was assayed as previously described [26]. Briefly, cell transfection was performed using TransIT-2020 transfection reagents according to the manufacturer’s instructions. LNCaP cells were plated at 10,000 cells per well and treated for 1 day with the indicated concentration of compound with increasing concentrations (0−25 μM) of compounds for screening and measuring IC_50_. Myc reporter activity was measured using the Cignal Myc Reporter Assay Kit from Qiagen (#336841; Hilden, Germany) according to the manufacturer’s instructions.

#### 4.2.2. Cell Viability Assays

NCI-H660 cells were purchased from the American Type Culture Collection (ATCC) and grown in RPMI medium supplemented with 5% fetal bovine serum (FBS), 1% Isulin-Transferrin-Selenium (Thermo Fisher, 41400-045, Waltham, MA, USA), 10 nM b-estradiol (Sigma, E8875; Darmstadt, Germany), 10 nM hydrocortisone (Sigma H0888), and 1% matrigel. HO15.19 cells were a generous gift from John Sidivy at Brown University and were cultured in Dulbecco’s modified Eagle’s medium (DMEM) (ATCC 30–2002) supplemented with 10% FBS. LNCaP-N-Myc and 22rv1-N-Myc cells were a generous gift from David Rickman at Weill Cornell Medicine (New York, NY, USA) and were cultured in RPMI supplemented with 10% FBS.

LNCaP were plated at 5000 cells per well in RPMI 1640 containing 5% CSS in a 96-well plate, treated with test compounds (0–25 μM) for 72 h. Cell density was measured using the PrestoBlue assay according to the manufacturer’s protocol. The percentage of cell survival was normalized to the cell density of control wells treated by vehicle.

Viability of N-Myc -overexpressing LNCaP and 22Rv1 was done with 5000 cells per well in RPMI supplemented with 10% FBS and read with Cell Titer Glo (Promega, cat# G7572; Madison, WI, USA) according to the manufacturer’s protocol. Eagle’s minimum essential medium (EMEM) supplemented with 10% FBS was used for the Myc-negative HO15.19 cells at 2000 cells per well and 20,000 neuroendocrine NCI-H660 cells were plated in RPMI supplemented with 5% FBS, 1% Isulin-Transferrin-Selenium (Thermo Fisher, 41400-045), 10 nM b-estradiol (Sigma, E8875), 10 nM hydrocortisone (Sigma H0888), and 1% matrigel. All cells were incubated for 72 h with the compounds.

#### 4.2.3. Aurora A Kinase Activity Assay

AURKA kinase assays were performed using the ADP-Glo Kinase assay (Promega) according to the manufacturer protocol. Measurement of Aurora-A activity was carried out using reactions containing (per well):10 ng Aurora-A catalytic domain, 50 uM ATP, and 0.5 myelin basic protein (MBP), in 5% dimethyl sulfoxide (DMSO). Reactions were incubated at room temperature for 60 min, and luminescence data (integration time: 0.5–1 s) was recorded on a Tecan M200 Pro reader (Männedorf, Switzerland).

#### 4.2.4. Biolayer Interferometry Assay

The direct interaction between biotinylated E-box oligo immobilized on a streptavidin biosensor and a purified N-Myc-Max complex (0.05 mg/ml) was quantified by BLI using OctetRED (ForteBio). The DNA was first bound to the super-streptavidin sensors. The sensors were next moved into wells containing the reaction buffer (20 mM Tris pH 8, 150 mM NaCl, 5% glycerol, 0.2 mM TCEP, 5% dimethylsulfoxide) for measuring the baseline and next into the N-Myc-Max complex alone or in presence of the tested inhibitors to study the association of the complex to the DNA.

## 5. Conclusions

In this study, our synergetic applications of CADD and experimental validation allowed us to design a novel dual-inhibitor, 70812, and to investigate the proposed N-Myc-Max DBD target site. We determined that the N′-[4-cyano-2-(trifluoromethyl)phenyl]-4-(trifluoromethyl)benzohydrazide scaffold is potent and selective against N-Myc, providing new insight into the proposed N-Myc-Max DBD pocket topology. As 70812’s tail is significantly more extended than 70551’s, the compound reveals the potential to explore novel therapeutics that are directed towards the N-terminus of the N-Myc-Max DBD pocket to enhance potency and specificity. 70812 exhibited low micromolar potency against PCa and NEPC cell lines by having inhibitory effects on both N-Myc and AURKA. We propose that 70812 blocks proliferation and growth of PCa and NEPC cells by interfering with N-Myc-Max interaction with DNA E-boxes through its N′-phenylbenzohydrazide scaffold, with AURKA’s kinase activity by blocking ATP from binding to its hinge region through its aminopyrazole-pyrimidine ATP-mimetic scaffold, and with AURKA’s ability to interact with N-Myc to stabilize it into an active form. Due to AURKA and N-Myc being extensively involved in the network of cell survival and growth, we report that effective inhibition of both targets could be a viable mechanism to design potent small molecules to treat NEPC patients. Consequently, the unique scaffold of 70812 represents a possible chemical starting point requiring extensive optimization for therapeutic development of a true dual-inhibitor targeting both N-Myc and AURKA directly with high potency and selectivity.

## 6. Patent

Compounds 70551 (PCT serial No.: PCT/CA2019/051243; filed 5 September 2019) and 70812 (US provisional patent serial No.: 62/985,275; filed 4 March 2020) can be made available to other researchers after standard Material Transfer Agreement (MTA) implementation with the University of British Columbia. 

## Figures and Tables

**Figure A1:**
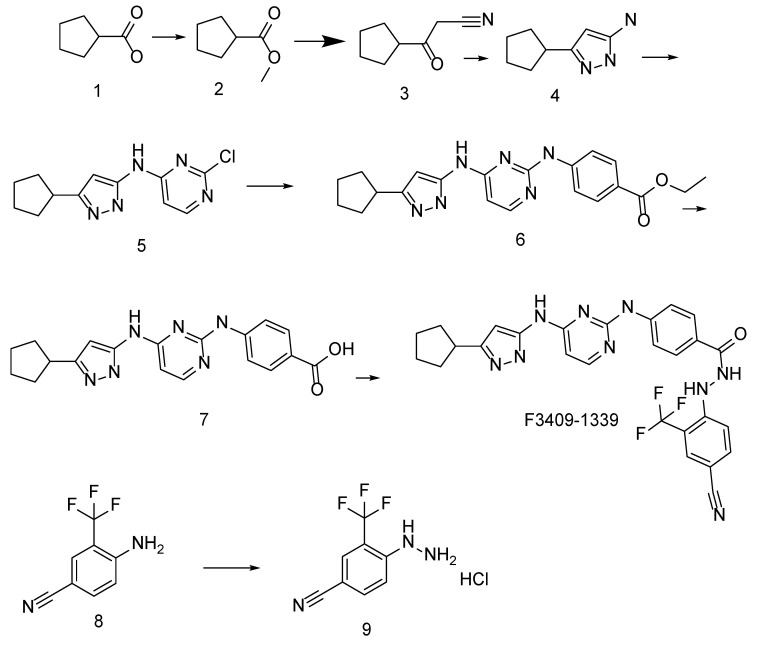
Synthetic Procedures for Synthesis of Compounds F3409-1339.

## Supplementary Materials

Supplementary materials can be found at https://www.mdpi.com/1422-0067/21/21/8277/s1.

## Abbreviations

AR	Androgen receptor
AURKA	Aurora A kinase
bHLHLZ	basic helix-loop-helix leucine zipper
BLI	bio-layer interferometry
CRPC	castration-resistant prostate cancer
DBD	DNA-binding domain
NEPC	neuroendocrine prostate cancer
PCa	prostate cancer
RMSD	Root-Mean Square Deviation
MD	Molecular dynamics

## Appendix A

**Compound 2.** Concentrated H_2_SO_4_ (7.35 g, 75 mmol) was added dropwise to a solution of **1** (50 g, 0.44 mol) in MeOH (500 mL) at room temperature. The reaction mixture was stirred under reflux for 10 h, the volatiles were removed in vacuo, the residue was neutralized with saturated solution of NaHCO_3_ and extract with DEE (2 × 250 mL). DEE was removed in vacuo and the residue was distilled *in vacuo* (1 tor) to give **1** (42 g, 75%).

**Compound 3.** MeCN (27 mL, 0.512 mol) was added dropwise to a suspension of NaH (20.5 g (0.512 mol, 60% in mineral oil) in anhydrous THF (170 mL). The reaction mixture was stirred at room temperature for 30 min and then compound **2** (42 g, 0.328 mol) was added dropwise thereto. The reaction mixture was stirred for 30 min at room temperature and then 16 h under reflux. The reaction mixture was cooled down to room temperature poured into cold water (10 °C) and unreacted starting materials were extracted by DEE (200 mL). The aqueous layer was acidified by HCl (3N) to pH 2 and extracted with DEE 2 × 200 mL. The organic layer was dried over Na_2_SO_4_ the dryer was filtered off washed with DEE and the solution was evaporated in vacuo to give 34 g of crude **3** which was used without further purification.

**Compound 4.** A solution of hydrazine hydrate (2 g, 40 mmol) and 2 (5.5 g, 40 mmol) in EtOH (20 mL) was stirred under reflux for 12 h. The reaction mixture was evaporated in vacuo and the residue was dissolved in CHCl_3_ (50 mL). The solution was washed with water (2 × 50 mL), dried over Na_2_SO_4_ the dryer was filtered off and the solution was evaporated *in vacuo*. The residue was recrystallized from EtOAc to give compound **4** (4.2 g, 69.3%).

**Compound 5.** A solution of compound **4** (1.32 g, 7.05 mmol), 2,4-dichloropyridazine (1 g, 6.71ммoль) and DPE (2.2 g, 16.8 mmol) in EtOH (10 mL) were stirred under reflux for 8 h. The reaction mixture was diluted with water (10 mL) the precipitate was filtered off and recrystallized from IPA to give 1.2g (67.8%) of **5**.

**Compound 6.** A solution of compound **5** (150 mg, 0.57 mmol), p-ethylaminobenzoate (90 mg, 0,6 mmol), Xantphos (44 mg), Pd(AcO)_2_ (30 mg), TEA (145 mg, 1.42 mmol) in dioxane (15 mL) was microwaved at 120 °C for 19 h. The precipitate was filtered off and washed with dioxane. The filtrate was evaporated the residue was purified on C18 reversed phase column to give **6** (50 mg, 22.4%)

**Compound 7.** A solution of NaOH (89 mg, 2.2 mmol) in water (1 mL) was added to a suspension of compound **6** (50 mg, 0.13 mmol) THF/H_2_O (1:1, 2 mL) and the reaction mixture was stirred at room temperature for 20 h, then neutralized with saturated solution of citric acid. The precipitate was filtered off, washed with water and dried to give **7** (40 mg, 86%).

**Compound 9.** A solution of NaNO_2_ (0.67 g, 9.7 mmol) in water (5 mL) was added dropwise to a suspension of **8** (1.5 g, 8.1 mmol) in concentrated HCl (10 mL) at 5 °C. The solution was filtered off and the filtrate was added dropwise to a vigorously stirred cooled (5 °C) suspension of SnCl2 (4.04 g, 17.9 mmol) in concentrated HCl (10 mL). The reaction mixture was stirred for 30 min and the precipitate was filtered off, suspended in cold water and was brought to pH = 10 by dropwise addition of aqueous NaOH solution (40%). The temperature must not be higher than 10 °C. The product was extracted with DEE (2 × 50 mL) the organic layer was dried over Na_2_SO_4_, the dryer was filtered off and washed with DEE. The filtrate was added dropwise to a saturated solution of HCl in dioxane (10 mL). The precipitate was filtered off washed with DEE and dried in vacuo to give **9** (1.31 g, 68.4%).

**Compound F3409-1339.** A solution of **7** (40 mg, 0.11 mmol) and CDI (21 mg, 0.13 mmol) in anhydrous DMF (0.2 mL) was stirred at 50 °C for 15 min, then hydrazine **9** (27 mg, 0.11 mmol) was added and the reaction mixture was stirred at 75 °C for 5 h. The final product was isolated from the reaction mixture via C18 reversed phase chromatography. Yield 20 mg (33.3%).
