# Supplementary material for: Dual-Inhibitors of N-Myc and AURKA as Potential Therapy for Neuroendocrine Prostate Cancer
Source: Int J Mol Sci. 2020 Nov 5;21(21):8277. doi: 10.3390/ijms21218277 (PMC7663809; doi:10.3390/ijms21218277)
Supplement: Supplementary file 1 [file ijms-21-08277-s001.pdf]

Table S1. Homology model evaluation.

PROSA Analysis	ID	Z-score (overall model quality)
	1NKP	-2.55
	MOE model	-2.44
**RMSD Analysis**	**ID**	**RMSD (Å)**
	MOE model	0.798 (88 atoms)

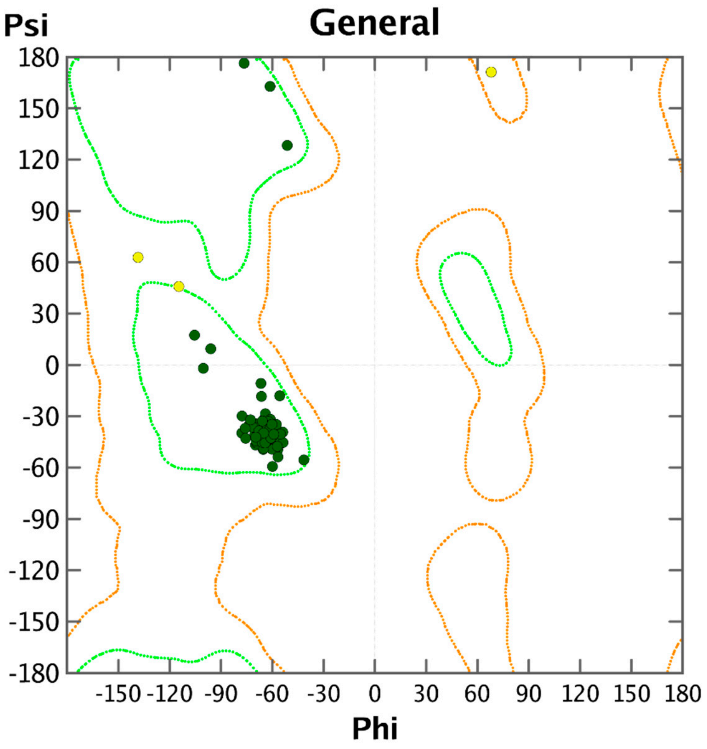

	No. of residues	Percentage
Most favoured regions	85	96.5%
Allowed regions	3	3.5%
Disallowed regions	0	0.0%
**Total number of residues**	**88**	**100.0%**

Figure S1. Ramachandran plot analysis of N-Myc homology model. Ramachandran plot of allowed φ/ψ distributions for the N-Myc homology model as determined in MOE. A good quality model would be expected to have over 90% of residues in the most favoured regions. Residues in the favoured regions are represented in green, while residues in the allowed regions are represented in yellow.

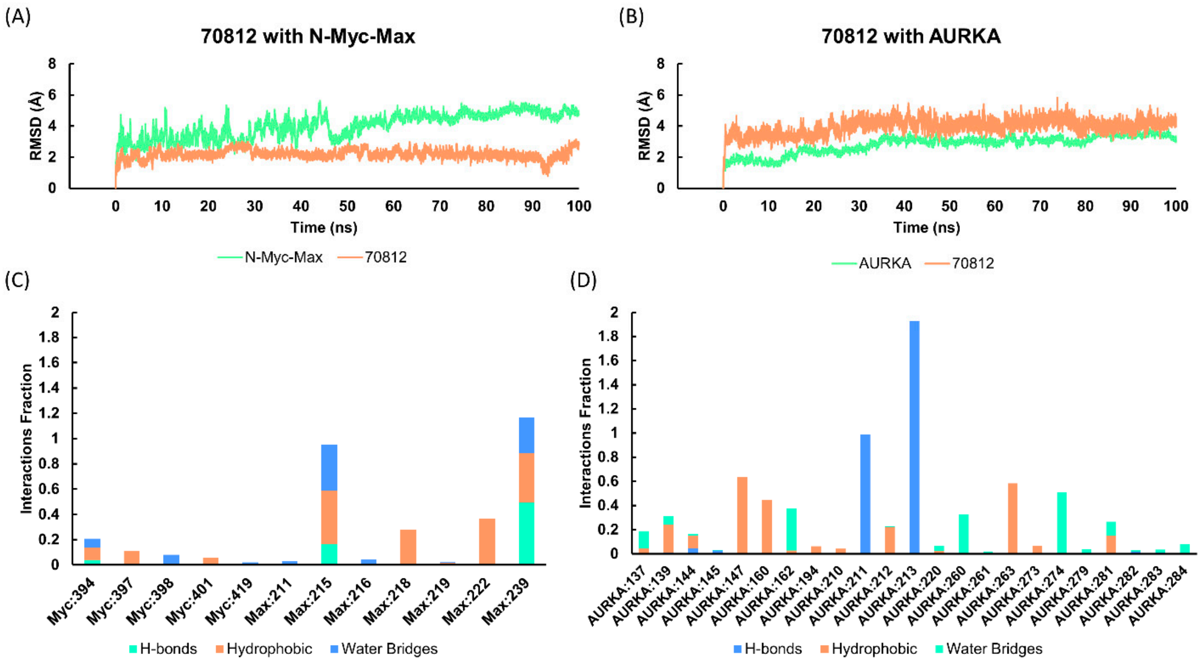

Figure S2. Molecular dynamics simulation analysis. (A) The N-Myc-Max-70812 complex was submitted to 100-ns MD simulations. The complex reach convergence after 50ns of simulations. RMSD calculations were carried out on Cα for N-Myc-Max and heavy atoms for 70812. Green: N-Myc-Max heterodimer; orange: 70812. **(B)** The AURKA-70812 complex was submitted to the same 100-ns MD simulations. The complex reached convergence after 35ns of simulations. RMSD calculations were carried out on Cα for AURKA and heavy atoms for 70812. Green: N-Myc-Max heterodimer; orange: 70812. **(C)** N-Myc-Max-70812 contacts chart. The interactions between the ligand and the protein were monitored throughout the simulations and are categorized by types: H-bonds (cyan), Hydrophobic (orange), and Water Bridges (blue). The charts are normalized over the full trajectory, but values over 1.0 represent protein residues that make multiple contacts with the ligand. **(D)** AURKA-70812 contacts chart.
